# Molecular chaperone Hsp27 regulates the Hippo tumor suppressor pathway in cancer

**DOI:** 10.1038/srep31842

**Published:** 2016-08-24

**Authors:** Sepideh Vahid, Daksh Thaper, Kate F. Gibson, Jennifer L. Bishop, Amina Zoubeidi

**Affiliations:** 1 The Vancouver Prostate Centre, University of British Columbia, Vancouver, British Columbia, Canada; 2 Department of Urologic Sciences, University of British Columbia, Vancouver, British Columbia, Canada

## Abstract

Heat shock protein 27 (Hsp27) is a molecular chaperone highly expressed in aggressive cancers, where it is involved in numerous pro-tumorigenic signaling pathways. Using functional genomics we identified for the first time that Hsp27 regulates the gene signature of transcriptional co-activators YAP and TAZ, which are negatively regulated by the Hippo Tumor Suppressor pathway. The Hippo pathway inactivates YAP by phosphorylating and increasing its cytoplasmic retention with the 14.3.3 proteins. Gain and loss of function experiments in prostate, breast and lung cancer cells showed that Hsp27 knockdown induced YAP phosphorylation and cytoplasmic localization while overexpression of Hsp27 displayed opposite results. Mechanistically, Hsp27 regulates the Hippo pathway by accelerating the proteasomal degradation of ubiquitinated MST1, the core Hippo kinase, resulting in reduced phosphorylation/activity of LATS1 and MOB1, its downstream effectors. Importantly, our *in vitro* results were supported by data from human tumors; clinically, high expression of Hsp27 in prostate tumors is correlated with increased expression of YAP gene signature and reduced phosphorylation of YAP in lung and invasive breast cancer clinical samples. This study reveals for the first time a link between Hsp27 and the Hippo cascade, providing a novel mechanism of deregulation of this tumor suppressor pathway across multiple cancers.

Heat shock proteins (HSPs) are highly conserved molecular chaperones with indispensable roles in protein homeostasis, transport processes and signal transduction. Although expressed under normal conditions, the expression of HSPs is upregulated in response to different cellular stresses including but not limited to, hyperthermia, hypoxia, oxidative stress and hormone- or chemo-therapy^1^,^2^. Thus, HSPs act as cytoprotective guardians, saving cells from stressors endangering their survival. In cancers however, these chaperones act as double-edged swords, where due to their active roles in protein homeostasis, HSPs enable survival of malignant cells. Among these upregulated HSPs in cancer is Hsp27 (HSPB1), an ATP independent small molecular chaperone that has been extensively studied as a result of its crucial involvement in diverse physiological and pathological cellular processes. Hsp27 is overexpressed across different cancers like prostate, breast, ovarian and gastric^3^,^4^,^5^,^6^,^7^,^8^ and contributes to cancer progression via different mechanisms; aside from its anti-apoptotic and pro-survival activities which play crucial parts in tumorigenesis^9^, Hsp27 increases proliferation by facilitating cell cycle progression^1^, and enhances migration and invasion via multiple mechanisms^10^,^11^. It also participates in the maintenance of cancer stem cells^12^,^13^. Moreover, overexpression of Hsp27 has been associated with poor prognosis and chemo-resistance in various cancers including prostate, breast, lung, cervical and bladder^14^,^15^,^16^,^17^,^18^.

The pleiotropic roles of Hsp27 underscore its position at hubs of cell signaling cascades across multiple cancers. Here, we show for the first time that Hsp27 is also involved in the Hippo tumor suppressor pathway, which restricts organ size and cell proliferation, and its inactivation correlates with poor patient outcome, increase of migration, invasion and metastasis^19^. Function of this evolutionarily conserved pathway requires a core kinase cascade, beginning with activation of MST1/2 (mammalian STE20-like protein kinases 1 and 2, with redundant roles)^20^ that in turn phosphorylate LATS1/2 (large tumour suppressor kinases 1 and 2) and MOB1 (adaptor proteins Mps one binder kinase activator 1). In normal cells, LATSs are responsible for phosphorylation of the transcriptional co-activators YAP1 (Yes associated protein) and its paralogue, TAZ (transcriptional co-activator with PDZ-binding motif) which results in their cytoplasmic sequestration with the 14.3.3 proteins and prevents their association with pro-tumor transcription factors^19^. In cancer however, reduced phosphorylation of YAP/TAZ allows for their translocation to the nucleus and activation of oncogenic and metastatic pathways, including TGF-β/SMAD, WNT/β-Catenin and ILK signaling^21^,^22^,^23^.

Here we demonstrate that Hsp27 is a negative regulator of the Hippo tumour suppressor pathway in human prostate, lung and breast cancer. We show for the first time that Hsp27 disrupts the Hippo kinase cascade, by accelerating the degradation rate of ubiquitinated-MST1 through the proteasome, which eventually lead to decreased YAP (S127) phosphorylation and its increased nuclear localization. Similarly, reduced levels of phospho-YAP (S127) were observed in human breast and lung tumors with higher Hsp27 expression. More importantly, we show for the first time that Hsp27 regulation of the Hippo pathway is reflected in the expression of YAP/TAZ target genes that are associated with aggressive cancer in prostate cancer cells as well as clinical prostate tumor samples. Taken together, our data point to the important role of Hsp27 in the inhibition of the Hippo tumour suppressor pathway in cancer cells, and further emphasize the significance of this molecular chaperone in human cancers.

## Results

### Identification of Hsp27 as a possible regulator of the Hippo tumour suppressor pathway

**Figure 1:**
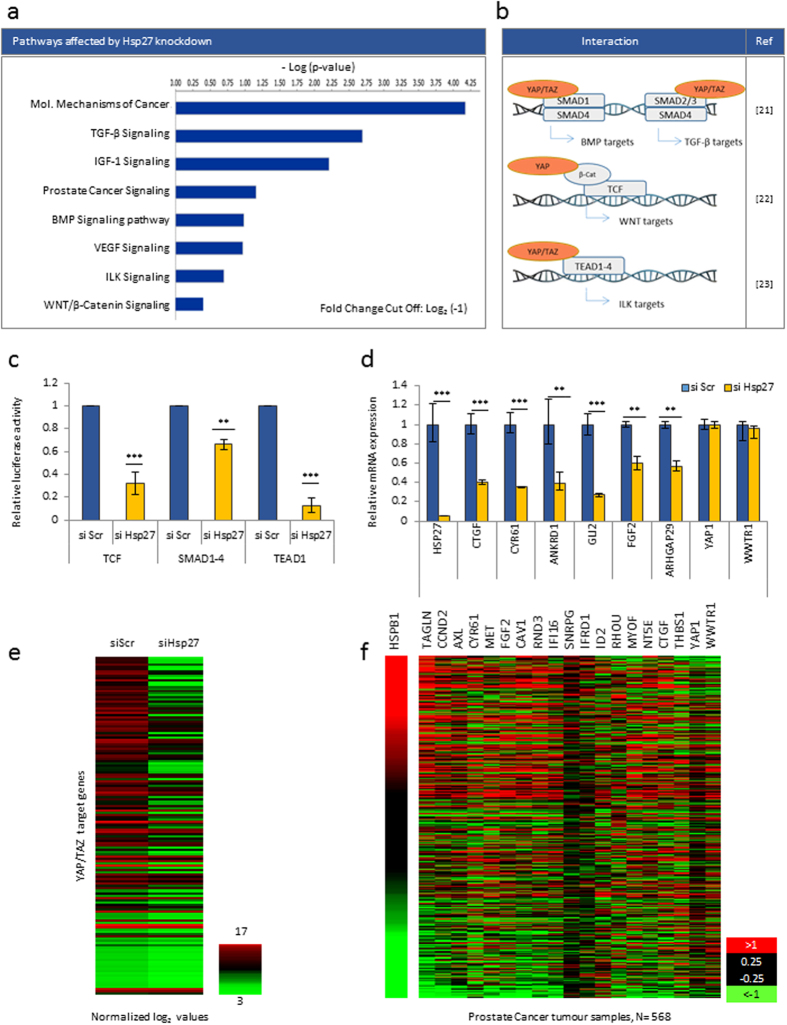
Hsp27 regulates YAP/TAZ transcriptional activity and downstream targets in Prostate Cancer. (**a**) IPA analysis of top pathways affected in si Hsp27 PC3 cells compared to si Scr. (**b**) Previously reported regulation of SMAD, TCF and TEAD1-4 by YAP/TAZ. (**c**) Relative activity of TCF, SMAD1-4 and TEAD1 assessed by luciferase assay in si Hsp27 PC3 cells compared to si Scr (=1), Graph represents pooled data from three independent experiments. (**d**) Relative mRNA expression of Hsp27 and YAP target genes in si Hsp27 PC3 cells compared to si Scr (=1). Graph is a representative of three independent experiments. (**e**) Microarray heat map showing expression of YAP/TAZ target genes (see Supplementary Table S1) in si Hsp27 PC3 cells compared to si Scr. Normalized log2 values were used to generate the heat map. (**f**) mRNA expression of Hsp27 (HSPB1) and YAP/TAZ target genes (see Supplementary Table S2) obtained from the Prostate Cancer TCGA data set.

Hsp27 is known to regulate multiple molecular pathways that contribute to prostate cancer progression; however an investigation of a global gene expression pattern regulated by Hsp27 in prostate cancer cells has not been reported. In order to identify novel pathways affected by Hsp27 expression, we performed a gene expression microarray in PC3 prostate cancer cells treated with small interfering RNA (siRNA) for Hsp27. Unbiased comparison of the gene expression signature in siScrambled (siScr) versus siHsp27 treated samples using Ingenuity Pathway Analysis (IPA) verified downregulation of major signaling pathways where Hsp27 has been shown to play a role (Fig. 1a). Importantly, as illustrated in Fig. 1b, the most highly downregulated signaling pathways, including TGF-β, BMP, WNT/β-Catenin and ILK all utilize YAP/TAZ as transcriptional co-activators^21^,^23^. However a link between YAP/TAZ and Hsp27 has not been reported. Confirming IPA pathway analysis results, we showed that the transcriptional activities of TCF, SMAD1-4 and TEAD1, well-established readouts of YAP/TAZ activity^22^,^24^,^25^, were significantly decreased in PC3 cells after Hsp27 knockdown compared to control (Fig. 1c). To further validate the regulation of YAP and TAZ by this chaperone, we tested the effect of siHsp27 on the expression of well-known YAP/TAZ target genes including CTGF, CYR61, ANKRD1, GLI2, FGF1 and ARHGAP29 in PC3 cells using qRT-PCR. Inhibition of Hsp27 resulted in significant down-regulation of these genes, but not YAP and TAZ themselves (Fig. 1d), suggesting that Hsp27 modulates YAP and TAZ activity but not their expression. Similar results were observed in lung cancer cell line A549 and breast cancer cell line MDA-Mb-453 where knocking down Hsp27 decreased YAP/TAZ transcriptional co-activity and their established target genes (Supplementary Figure S1b,c).

To further investigate the impact of Hsp27 on YAP/TAZ targets, a comprehensive list of YAP/TAZ target genes was compiled^26^,^27^,^28^ (Supplementary Table S1) and microarray expression in siScr versus siHsp27 treated PC3 cells was compared. In accordance with our initial results, more than 70% of YAP/TAZ targets were down-regulated in siHsp27 samples compared to control (Fig. 1e). These *in vitro* findings were supported by clinical data showing that expression of Hsp27 (HSPB1) and a subset of these YAP/TAZ target genes (Supplementary Table S2) were positively correlated in the publicly available TCGA database for prostate cancer tissues^29^ (Fig. 1f, The Cancer Genome Atlas, https://genome-cancer.ucsc.edu/). Taken together, our data suggest that Hsp27 knockdown decreases the activity of YAP/TAZ cooperating transcription factors leading to a downregulation of their targets, a hypothesis supported by both *in vitro* and human cancer data.

### Hsp27 is required for YAP activation and nuclear translocation in cancer cells

**Figure 2:**
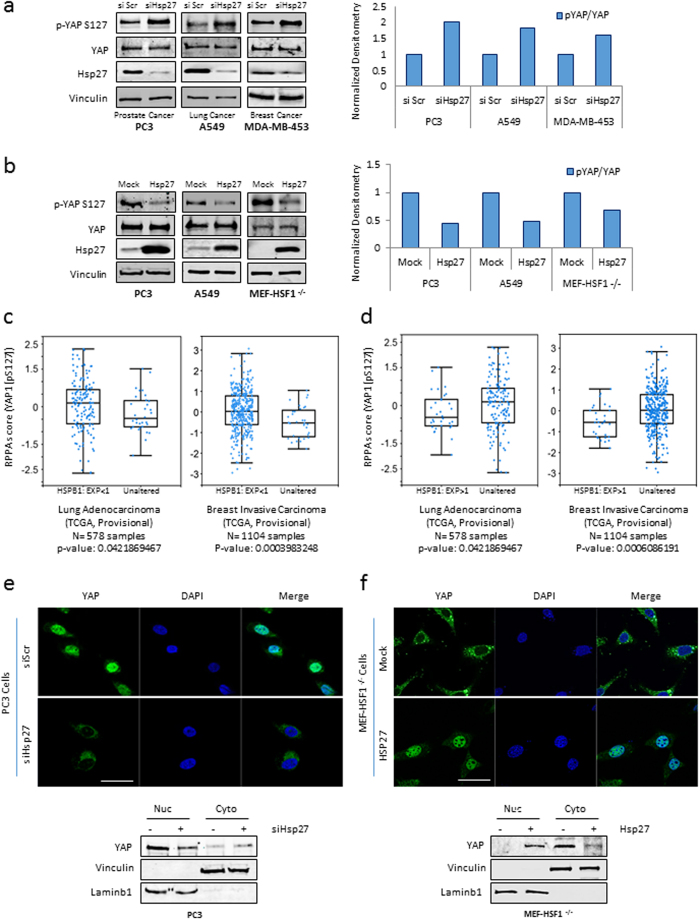
Hsp27 is required for YAP activation and nuclear translocation. (**a,b**) Protein expression of p-YAP S127, YAP, Hsp27 and Vinculin in PC3, A549, MDA-MB-453 and/or MEF-HSF1^−/−^ cells transfected with (**a**) si Hsp27 or si Scr or (**b**) Hsp27 or vector control (mock). Densitometry shows fold expression of p-YAP S127 compared to total YAP (=1). (**c,d**) RPPA score of p-YAP S127 in Lung Adenocarcinoma and Invasive Breast Carcinoma in tumors with Hsp27 (HSPB1) expression (**c**) less than 1 or (**d**) more than 1 z-score obtained from TCGA data sets. (**e**,**f**) Immunofluorescence of total YAP (green) and the nucleus (DAPI, blue) in PC3 or MEF-HSF1^−/−^ cells transfected with (**e**) si Hsp27 or si Scr or (**f**) Hsp27 or vector control (mock). Scale bar: 50 μm. Lower western blots show nuclear and cytoplasmic levels of total YAP, vinculin and lamin B1 in knockdown and overexpression of Hsp27.

Our results suggested that Hsp27 affects YAP activity without affecting its transcription (Fig. 1d). Post-translationally, YAP activity is inhibited by phosphorylation on S127 which prevents its nuclear translocation^19^. As shown in Fig. 2a, siRNA inhibition of Hsp27 lead to increased S127 phosphorylation of YAP in PC3 prostate, A549 lung and MDA-MB-453 triple negative breast cancer cells. This was validated using different commercially available siRNAs (Supplementary Figure S1a)^30^. Testing the transcription capacity of TCF, SMAD1-4 and TEAD1 in A549 and MDA-MB-453 cells, we further demonstrated that S127 phosphorylation decreases YAP activity (Supplementary Figure S1b). Moreover, similar to what we observed in PC3 cells, YAP/TAZ target genes decreased upon Hsp27 knockdown in both cell lines (Supplementary Figure S1c). Reciprocally, Hsp27 overexpression decreased the inhibitory phosphorylation of YAP on S127 in PC3 and A549 cells compared to control (Fig. 2b). We further tested this phenomenon using a mouse embryonic fibroblast cell line that lack heat shock factor protein 1 (HSF1) (MEF-HSF1^−/−^), a transcription factor required to initiate transcription of all HSPs; these cells therefore do not express Hsp27 and provide a useful model to study the effects of Hsp27 overexpression on the Hippo pathway. In concordance with our observations in PC3 and A549 cells, phosphorylation of YAP on S127 decreased upon Hsp27 transfection (Fig. 2b) in MEF-HSF1^−/−^ cells.

To validate the clinical relevance of our findings, we interrogated the RPPA (reverse phase protein lysate microarray) score of p-YAP S127 in Hsp27 overexpressing tumors found in the cBioportal repository (www.cbioportal.org)^31^. Figure 2c illustrates statistically significant upregulation of p-YAP S127 in lung (Fig. 2c left) and breast (Fig. 2c right) tumor samples that have lower Hsp27 expression (EXP < 1) compared to samples with unaltered Hsp27. The opposite trend was observed in tumors that have higher Hsp27 expression (EXP > 1); lung (Fig. 2d left) and breast (Fig. 2d right) cancer tissue samples with overexpression of Hsp27 showed significantly lower phosphorylation on YAP S127. These data combined with our *in vitro* findings further support a role for Hsp27 in the negative regulation of the Hippo pathway in human cancer.

Phosphorylation of YAP on S127 prevents its translocation to the nucleus^19^. Based on our results showing Hsp27 affects p-YAP levels, we investigated the localization of YAP upon changes in Hsp27 expression. Interestingly, immunofluorescence showed extensive cytoplasmic localization of YAP in PC3 upon Hsp27 knockdown compared to siScr treated cells, in which YAP remained mostly nuclear (Fig. 2e top). This phenomenon was also captured by western blot (Fig. 2e bottom). Reciprocally, in MEF-HSF1^−/−^ cells, immunofluorescence showed nuclear localization of YAP increased after Hsp27 overexpression compared to mock transfected cells (Fig. 2f top), and cytoplasmic/nuclear fractionation of MEF-HSF1^−/−^ cells clearly demonstrated the translocation of YAP upon Hsp27 overexpression (Fig. 2f bottom). Taken together, these findings indicate that expression of Hsp27 controls YAP activity by affecting its phosphorylation and subsequent nuclear translocation.

**Figure 3:**
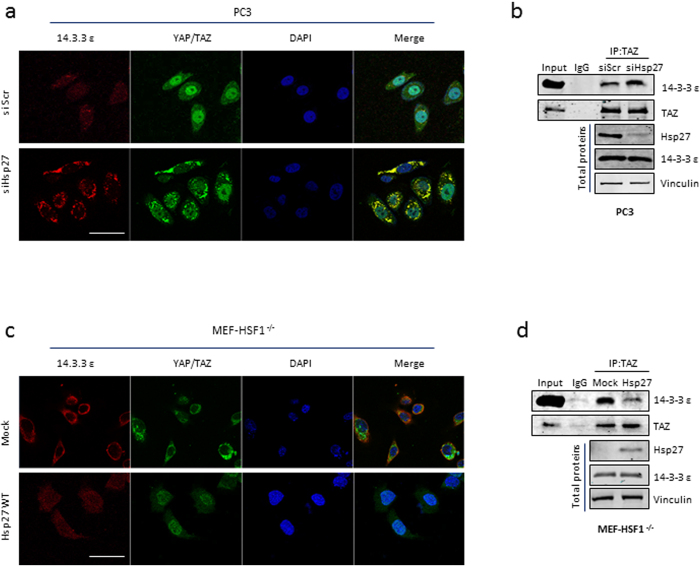
Hsp27 regulates cytoplasmic sequestration of YAP/TAZ. (**a**,**c**) Immunofluorescence of 14.3.3 (red), YAP/TAZ (green) and the nucleus (DAPI, blue) in PC3 or MEF-HSF1^−/−^ cells transfected with (**a**) si Hsp27 or si Scr or (**c**) Hsp27 or vector control (mock). Scale bar: 50 μm (**b**,**c**) Protein expression of 14.3.3 ε, total TAZ, Hsp27 and vinculin after TAZ immunoprecipitation in PC3 or MEF-HSF1^−/−^ cells transfected with (**b**) si Hsp27 or si Scr or (**d**) Hsp27 or vector control (mock).

Phosphorylation of YAP and its paralogue TAZ lead to their sequestration in the cytoplasm by the 14.3.3 proteins^19^. Concordantly, we observed that Hsp27 silencing resulted in not only increased cytoplasmic sequestration, but also in significant co-localization of YAP/TAZ with 14.3.3 in PC3 cells (Fig. 3a). An increase in interaction between 14.3.3 and TAZ was also observed upon Hsp27 siRNA treatment (Fig. 3b). Reciprocally, YAP/TAZ were more nuclear and less sequestered with the 14.3.3 proteins upon Hsp27 overexpression in MEF-HSF1^−/−^ cells (Fig. 3c). This decreased interaction between TAZ and 14.3.3 proteins was also observed by immunoprecipitation in MEF-HSF1^−/−^ cells after Hsp27 overexpression (Fig. 3d). Together, these findings suggest that Hsp27 regulates YAP and TAZ activity through inhibition of phosphorylation, decreasing their sequestration by 14.3.3 proteins and promoting their translocation to the nucleus.

### Hsp27 regulates the Hippo pathway by affecting core kinase components

**Figure 4:**
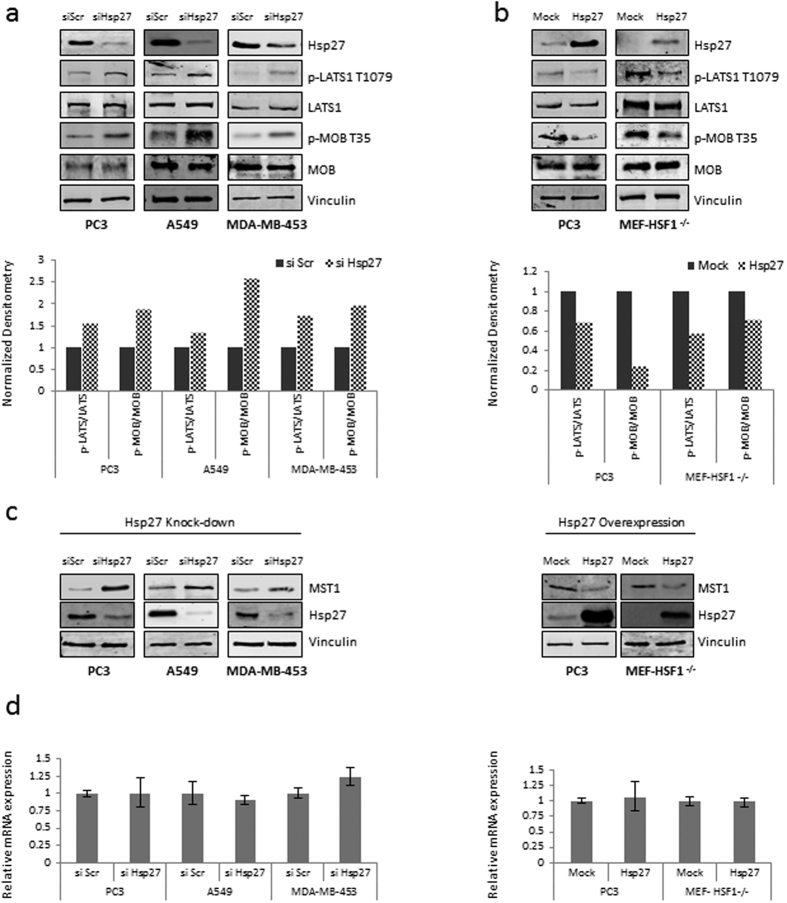
Hsp27 negatively regulates the Hippo pathway. (**a,b**) Protein expression of Hsp27, p-LATS1 T1079, total LATS1, p-MOB T35, total MOB1 and Vinculin in PC3, A549, MDA-MB-453 and/or MEF-HSF1^−/−^ cells transfected with (**a**) si Hsp27 or si Scr or (**b**) Hsp27 or vector control (mock). Densitometry shows fold expression of p-LATS1 T1079 compared to total LATS1 (=1) and p-MOB1 T35 compared to total MOB1 (=1). (**c**) Protein expression of Hsp27, MST1 and vinculin in PC3, A549, MDA-MB-453 and/or MEF-HSF1^−/−^ cells transfected with si Hsp27 (left panel) or Hsp27 plasmid (right panel) compared to control (si Scr or Mock transfected cells). (**d**) Relative mRNA expression of MST1 in PC3, A549, MDA-MB-453 and/or MEF-HSF1^−/−^ cells transfected with si Hsp27 (left graph) or Hsp27 plasmid (right graph) compared to control (si Scr or Mock transfected cells = 1). Graphs are representatives of three independent experiments.

Our results indicate that Hsp27 modulates YAP phosphorylation status, which is controlled by the Hippo pathway through a core kinase cascade involving LATS1, MOB1 and MST1^19^; however a relationship between these kinases and Hsp27 has not been described yet. To examine the effect of Hsp27 in regulating the phosphorylation of YAP in more detail, PC3, A549 and MDA-MB-453 cells were treated with Hsp27 siRNA and functional changes in the Hippo pathway kinase cascade were examined. YAP is directly phosphorylated and restrained by LATS1, which is active when it is phosphorylated on T1079^32^. As shown in Fig. 4a, knocking down Hsp27 resulted in increased p-LATS1 (T1079) across three cell lines, while the total protein remained unchanged. Looking further upstream in the Hippo pathway, we observed that phosphorylation of MOB1 on T35, which is required for activation of LATS1^33^, was also increased in siHsp27 treated samples compared to siScr (Fig. 4a). Reciprocally, we observed that Hsp27 overexpression In PC3 and MEF-HSF1^−/−^ cells drastically decreased phosphorylation of LATS1 (T1079) and MOB1 (T35) (Fig. 4b).

Finally, we examined the effects of Hsp27 modulation on MST1, the mammalian homologue of Drosophila’s Hippo kinase that is directly responsible for phosphorylation of LATS1 and MOB1^33^. In accordance with changes observed in pLATS1 and pMOB1, we found that loss of Hsp27 increased MST1 total protein (Fig. 4c left). Reciprocally, overexpression of Hsp27 decreased total MST1 across multiple cell lines (Fig. 4c right). Importantly, no changed was observed in mRNA levels of MST1 (Fig. 4d), and overexpression of MST1 did not affect Hsp27 while increasing p-YAP and subsequently decreasing its co-activator function (Supplementary Figure S2a,b). Taken together, these results indicate that Hsp27 inhibits the Hippo tumor suppressor pathway by regulating MST1 at the protein level.

### Hsp27 forms a complex with ubiquitinated MST1 and accelerates its proteasomal degradation

**Figure 5:**
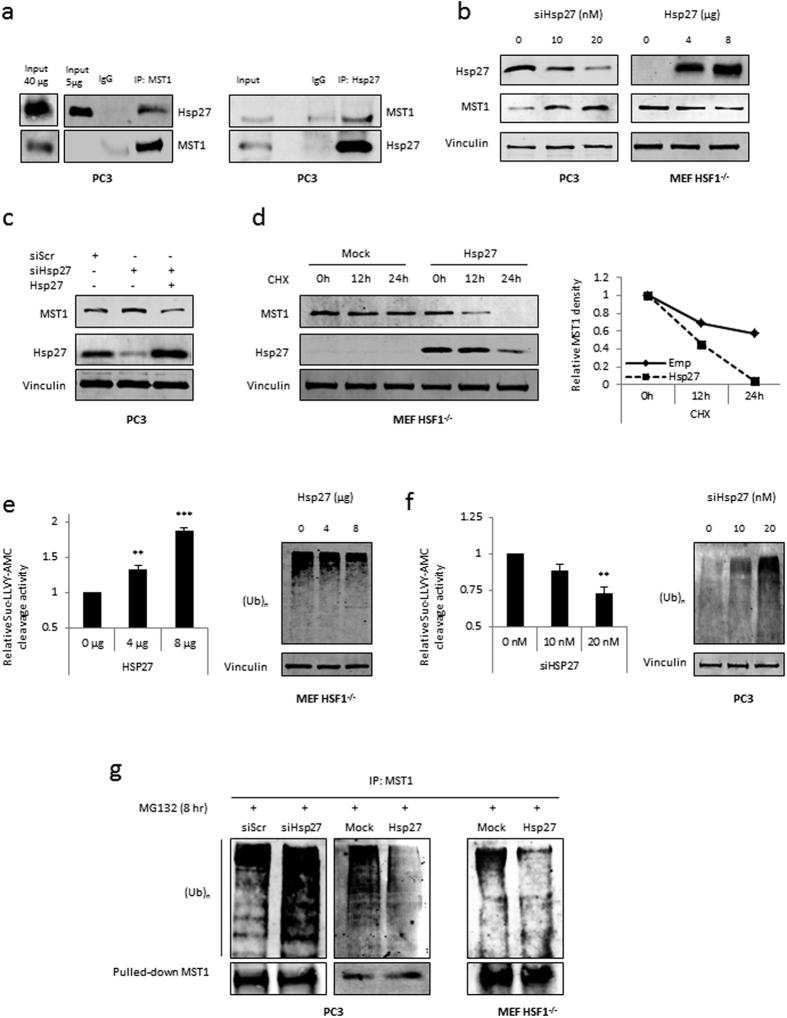
Hsp27 forms a complex with and increase the degradation rate of MST1. (**a**) Protein expression of Hsp27, MST1 and IgG after MST1 (right) and Hsp27 (left) immunoprecipitation in PC3 cells. (**b**) Protein expression of Hsp27, MST1 and vinculin in PC3 and MEF-HSF1^−/−^ cells transfected with increasing dose of si Hsp27 (left panel) or Hsp27 plasmid (right panel). (**c**) Protein expression of Hsp27, MST1 and vinculin upon rescuing Hsp27 after treatment with siRNA in PC3 cells. (**d**) Rate of degradation for MST1 in the presence and absence of Hsp27 when *de novo* protein synthesis is inhibited using 10 μg/ml of Cyclohexamide (CHX) at different time points. Western blot shows protein expressions of Hsp27, MST1 and vinculin in MEF-HSF1^−/−^ cells transfected with Hsp27 plasmid. Levels of MST1 were quantified using densitometry. (**e,f**) Proteasome activity was monitored for cleavage of the Suc-LLVY-AMC substrate and the expression of ubiquitinated proteins were analyzed using ubiquitin antibody. Graph represents pooled data from three independent experiments. (**e**) PC3 cells with increasing doses of Hsp27 siRNA. (**f**) MEF-HSF1^−/−^ cells transfected with increasing dose of Hsp27. (**g**) Protein expression of ubiquitin and MST1 after MST1 immunoprecipitation in si Hsp27 (right) or Hsp27 plasmid (middle) transfected PC3 cells as well as Hsp27 plasmid transfection in MEF-HSF1^−/−^ cells (right panel) treated with 10 μM MG132 (8 hr).

In an effort to understand the exact mechanism by which Hsp27 regulates protein levels of MST1, we unveiled for the first time that endogenous MST1 and Hsp27 form a complex together (Fig. 5a, left) which was further confirmed with the reciprocal immunoprecipitation (Fig. 5a, right). The effect of Hsp27 on MST1 was observed in a dose-dependent manner after Hsp27 knockdown and overexpression (Fig. 5b). In addition, rescuing the expression of Hsp27 reduces MST1 levels that were increased by Hsp27 siRNA treatment (Fig. 5c). We then examined if Hsp27 regulates the stability of MST1, by studying the rate of degradation for MST1 in the presence or absence of Hsp27 and the protein synthesis inhibitor Cycloheximide (CHX). Our results showed that MST1 degrades drastically faster in the presence of Hsp27 in MEF-HSF1^−/−^ cells (Fig. 5d). Interestingly, in harmony with prior reports^34^,^35^, this effect correlates with a dose dependent increase in the proteasome activity along with reduced accumulation of ubiquitinated proteins upon Hsp27 overexpression compared to control (Fig. 5e). On the other hand, knocking down Hsp27 decreased the proteasome activity and accumulated ubiquitinated proteins (Fig. 5f).

Consistent with these results, and previous reports showing MST1 degradation is ubiquitin-mediated^36^, we found that in the presence of the proteasome inhibitor MG132, Hsp27 knockdown increased levels of ubiquitinated MST1 while overexpression of Hsp27 decreased it (Fig. 5g, left). Similarly, a decrease in ubiquitinated MST1 was observed in MEF-HSF1^−/−^ cells upon introduction of Hsp27 (Fig. 5g, right). Importantly, these results were independent of Hsp70, which was previously shown to increase ubiquitination of MST1^37^, as no change in Hsp70 was observed in Hsp27 knockdown or overexpression experiments nor in Hsp90 or clusterin (Supplementary Figure S3a,b). The unique role of Hsp27 as a strong modulator of MST1 independent of Hsp70 is supported by human data, which showed that in contrast to Hsp27, Hsp70 expression in human PCa samples does not correlate with YAP/TAZ gene signature (Supplementary Figure S3c). Taken together, these findings suggest an independent role for Hsp27 in MST1 regulation by forming a complex with and accelerating the degradation rate of ubiquitinated MST1.

**Figure 6:**
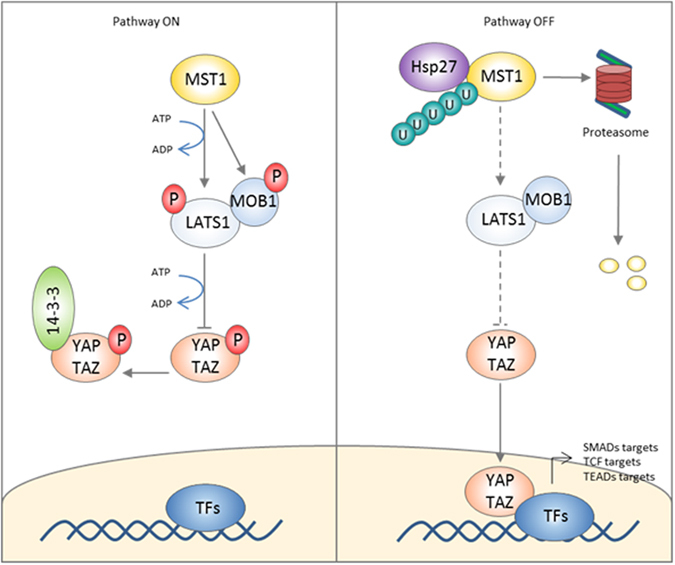
Proposed mechanism of Hsp27 regulation of the Hippo tumor suppressor pathway. (**Pathway On**) The Hippo tumor suppressor pathway is activated by the core kinase MST1, which phosphorylates LATS1 and MOB1, leading to YAP/TAZ phosphorylation and their sequestration in the cytoplasm by 14.3.3 proteins. This prevents YAP/TAZ nuclear translocation and association with transcription factors such as SMADs, TCF and TEADs. (**Pathway Off**) Hsp27 forms a complex with MST1 and accelerates the proteasomal degradation of ubiquitinated MST1. This prevents phosphorylation of LATS1 and MOB1, and phosphorylation of YAP/TAZ, which allows their nuclear translocation and their transcriptional activation of genes associated with malignant phenotypes.

Based on our accumulated *in vitro* data, we propose that Hsp27 overexpression in cancer cells inactivates the Hippo tumor suppressor pathway by potentiating MST1 degradation, resulting in the disruption of the Hippo kinase cascade, decreased YAP phosphorylation, and ultimately increased YAP/TAZ nuclear localization that drives transcription of genes associated with malignant cell phenotypes (Fig. 6).

## Discussion

The ability of cancer cells to survive in an environment filled with stress inducers depends on how they respond to these stimuli; In the context of such toxic stress, cancer cells activate survival pathways including overexpression of heat shock proteins, especially Hsp27. Extensive investigation of Hsp27 expression in human tumors has shown that Hsp27 is elevated across different cancers and is associated with aggressive and treatment-resistant malignancies^1^. Research driven by our laboratory and others showed that Hsp27 promotes cell proliferation and tumor growth in different cancers including prostate and bladder^35^,^38^ and that targeting Hsp27 not only reduces tumor progression, but also opposes chemotherapy resistance in prostate and lung cancer^35^,^39^,^40^. Moreover, we showed that Hsp27 drives β-catenin-mediated Epithelial Mesenchymal Transition (EMT) that is required for invasion and metastasis in prostate cancer^10^,^11^. Numerous studies emphasize on the essential part Hsp27 plays in malignancies; however, a global gene expression pattern regulated by this chaperon in prostate cancer cells was not investigated.

Using an un-biased approach, we found for the first time that transient knockdown of Hsp27 reduces the gene signature of several cellular pathways including WNT/β-catenin, TGF-β/SMADs and ILK signaling. Detailed examination of gene transcription activities in these pathways revealed that they all share YAP and TAZ as transcriptional co-activators^21^,^22^,^23^. The onco-proteins YAP and TAZ intertwine closely with cellular pathways and are key modulators in organ size, cell proliferation and apoptosis. Interestingly, increased expression of YAP/TAZ, their nuclear localization, as well as elevation of their target genes, are reported in many types of cancers and are known to be involved in malignant phenotypes resulting in enhanced cell proliferation, EMT and drug resistance^19^. For example, activation of YAP is highly associated with poor prognosis and treatment resistance in colorectal cancer^41^ and promotes migration and invasion in prostate cancers cells^42^. In addition, nuclear TAZ is present in 90% of metaplastic (a subtype of triple negative) breast cancers with EMT morphology^43^. YAP and TAZ have also been associated with progression and metastasis in lung cancer^44^ and their direct transcription targets, CTGF and AXL, were linked to EMT and drug resistance in this disease^45^.

YAP/TAZ activity and protein levels are predominantly controlled by the Hippo tumor suppressor pathway. First discovered in *Drosophila melanogaster*, the Hippo pathway has a fundamental role in organ growth control, stem cell function, regeneration and tumour suppression and its decreased activity is associated with poor outcome across multiple cancer types^46^. The Hippo signaling has been shown to cross talk with a number of other molecular pathways commonly altered in human cancers such as WNT and TGF-β pathway^47^. Similarly, Hsp27 is recognized as a central hub connecting networks that promote tumor growth and progression^1^. While there is extensive overlap between the cell survival, anti-apoptotic and metastatic pathways that Hsp27 and the Hippo components may regulate, a relationship between the two has not been described yet. Here, for the first time, we report that Hsp27 negatively regulates the Hippo tumor suppressor pathway across different cancers.

Our research indicates a positive correlation between Hsp27 expression and YAP/TAZ activity. Interestingly, this positive correlation to YAP/TAZ gene signature was also observed in the TCGA data of prostate cancer tumors, suggesting the clinical relevance of this association. Mechanistic investigations revealed that Hsp27 regulates YAP by decreasing the inhibitory phosphorylation of YAP on Serine 127. Strikingly, patient data analysis of lung and breast adenocarcinomas from TCGA demonstrated the same pattern where tumors with higher expression of Hsp27 showed lower p-YAP (S127) providing further evidence that Hsp27 regulates YAP activity. Our data suggest that the functional consequence of overexpression of Hsp27 and subsequent decreased phosphorylation of YAP, is the increased nuclear translocation of this transcription co-activator, allowing it to drive transcription of pro-tumorogenic genes^48^. Most importantly, we report for the first time that the effects on YAP phosphorylation are dependent on Hsp27 negative regulation of the core Hippo kinase, MST1.

MST1 (STK4) is a multifunctional kinase with tumor suppressive roles and is considered an independent prognostic factor in different cancers, where its reduction or loss of expression is associated with poor prognosis^49^,^50^,^51^. A study on more than 1000 colorectal cancer samples showed that loss of cytoplasmic MST1 was an independent adverse prognostic factor in this disease^49^. In addition, MST1 protein, but not mRNA, expression in prostate adenocarcinoma is significantly downregulated compared to paired normal tissue^52^, pointing to the importance of post-translational modifications of this kinase in prostate cancer. Another independent study revealed that levels of MST1 also decreased with progression of the disease to the more aggressive form, castration resistant prostate cancer (CRPC)^51^. These findings, combined with our previous work showing that transition from hormone naïve PCa to CRPC is accompanied by an increase in the expression of Hsp27^11^, suggest that Hsp27 may control MST1 in CRPC progression.

Furthermore, our study provides mechanistic insight into how MST1 levels are decreased in aggressive cancers. Although previous work in colon cancer suggested post-translational control of MST1^49^, little else is known about the underlying mechanism by which MST1 is reduced, except that its degradation is ubiquitin-mediated^36^,^37^. The ubiquitination system brands proteins to be degraded by the proteasome. Interestingly, containing an ubiquitin-like domain, Hsp27 directly binds to one of the subunits of 26S proteasome and enhances the catalytic activity of the 26S proteasome machinery in response to stressful stimuli. Here for the first time, we show that Hsp27 forms a complex with MST1 and enhances its rate of proteasomal degradation. This finding is in concordance with previous studies describing the role of Hsp27 in enhancing the degradation of anti-proliferative proteins, I-κB (inhibitor of kappa B) and p27^Kip1^ (cyclin-dependent kinase inhibitor)^34^,^53^. Moreover, our data compliments previous work which showed MST1 interacts with the ATPase domain of Hsp70 and that overexpression of Hsp70 in stress conditions increases ubiquitination of MST1 in PCa cells^37^. Although Hsp27 and Hsp70 share several properties in the control of protein homeostasis, they are indeed different in terms of how they regulate these processes. For example, unlike Hsp70, Hsp27 is an ATP independent chaperone. In addition, the enhancement of proteasomal activity by Hsp70 in response to stress, does not depend on a direct interaction with ubiquitin chains, as it is the case with Hsp27^54^. Comparing our results to those involving Hsp70 highlights that these molecular chaperones independently regulate two important steps in the ubiquitination (Hsp70) and subsequent degradation (Hsp27) of MST1, underscoring their complementary and essential roles in controlling the Hippo cascade.

In summary, the novel link our data establish between Hsp27 and the Hippo pathway adds to the mechanisms by which Hsp27 may control tumor development and phenotype. For the first time we show that Hsp27 increases degradation rate of ubiquitinated MST1 and therefore interrupts the Hippo pathway kinase cascade. Consequently YAP and TAZ onco-proteins are less phosphorylated, free to translocate into the nucleus and promote a malignant phenotype (Fig. 6). These findings, as well as corresponding clinical correlations we found in multiple tumor types underscore the central importance of Hsp27 in regulating multiple signaling pathways that promote tumor aggressiveness and suggest that especially in tumor types that depend heavily on inactivation of the Hippo pathway, inhibition of Hsp27 may be efficacious in preventing tumor progression.

## Materials and Methods

### Cell culture and transfection

Prostate cancer PC3 and lung cancer cell line A549 were purchased from the American Type Culture Collection (ATCC) and authenticated by isoenzymes analysis in 2008 and short tandem repeat (STR) profile in 2013 respectively. Both cell lines were maintained in RPMI media supplemented with 10% fetal bovine serum (FBS, Invitrogen-Life Technologies). Breast cancer cell line MDA-MB-453 (ATCC) and HSF1 knock-out murine embryonic fibroblasts (MEF HSF1^−/−^) (a kind gift from I. J. Benjamin, University of Utah) were maintained in Dulbecco’s modified Eagle’s medium (DMEM; Invitrogen Life Technologies) containing 10% FBS. For small interfering RNA transfection, PC3, A549 and MDA-MB-453 were transfected with 20 nM Hsp27 siRNA (siHsp27) or control siRNA (siScr) using OligofectAMINE (Invitrogen-Life Technologies, Inc.) in Opti-MEM (Gibco) following the manufacturer procedures. pHR-CMV Empty and wild type Hsp27 vectors (WT) were used as previously described^9^. Cells were seeded at 5 × 10^5^ cells and 10^6^ cells in 10 cm^2^ dishes for siRNA and plasmid transfections respectively.

### Western blotting and immunoprecipitation

RIPA lysis buffer with protease and phosphatase inhibitors were used for total proteins extraction as previously described^30^. Nuclear/cytoplasmic fractions were extracted using the CelLytic^TM^ NuCLEAR^TM^ Extraction Kit (Sigma-Aldrich, St Louis, MO) according to manufacturer’s protocol. Immunoprecipitation was performed using ImmunoCruz™ IP/WB Optima B System (Santa Cruz) based on the manufacturer’s guideline. 2 μg of primary antibody, or immunoglobulin G (IgG) was used for immunoprecipitation and control respectively. Blots were incubated overnight at 4 °C with designated primary antibodies at 1:1000 dilution, unless noted otherwise. Proteins were visualized using the Odyssey system (Li-Cor Biosciences) and densitometric analysis was performed using ImageJ software (National Institutes of Health, USA).

### Reagents and antibodies

Antibodies against YAP (#4912), YAP/TAZ (#8418), phospho-YAP S127 (#4911), LATS1 (#3477, 1:200), phospho-LATS1 Thr1079 (#9159, 1:200), MOB1 (#3863), phosphor-MOB1T35 (#8699), MST1 (#3682) were purchased from Cell Signaling, USA, anti-14:3:3 ε (sc-393177), TAZ (sc-48805), Ubiquitin (sc-8017) and Lamin B1 (sc-377001) were from SantaCruz Biotechnology. Anti-LATS1 ab70562 (abcam) and anti-MOB1A/B sc-161867 (Santa Cruz) were used in the mouse cell line with 1:500 dilution. Anti-Hsp27 (1:5000) was from ENZO lifesciences. MG132 was from Millipore (#474791).

### Immunofluorescence

PC3 and MEF HSF1^−/−^ cells were grown and double transfected with siScr/siHsp27 and mock/Hsp27 respectively, in 10 cm^2^ plates. Then the cells were trypsinized and plated in 12 well plates at 5000 cells/cm^2^ on coverslips submerged in RPMI+FBS 10% for 24 hours. Cells were then fixed and the Immunofluorescence was performed as we previously reported^30^ using antibodies against YAP/TAZ (1:500) and 14.3.3 epsilon (1:200). DAPI was used to visualize nuclei and then the pictures were taken by 63x objective lens using Zeiss LSM confocal microscope. Results are representative of random pictures taken from three independent experiments.

### Quantitative reverse transcription (RT)-PCR

TRIzol^®^ reagent (Invitrogen) was used to extract total RNA from cultured cells. 2 μg of total RNA was reversed transcribed using random hexamers (Applied Biosystems) as previously reported^11^. Q-RT PCR amplification of cDNA was performed using the following primer pairs (sequences listed in Supplementary Table S3): Hsp27, YAP1, WWRT1, STK4, CTGF, CYR61, GLI2, FGF2, ARHGAP29, ANKRD1, GAPDH with FastStart Universal SYBR Green Master (ROX; Roche Applied Science) on the ABI ViiA7 Sequence Detection System. Target gene expression was normalized to GAPDH levels. The results represent three independent experiments with each sample ran in triplicate.

### Luciferase Assay

For TEAD, TCF and SMAD transcriptional activity, 2 × 10^4^ cells were plated in triplicate in six-well plates and transfections were carried out using TransIT^®^-2020 (Mirus Bio.) and 9 μg of indicated luciferase reporter for each 6 well plate: pGL3-OT for TCF, 8xGTIIC-luc for TEAD and SBE4-Luc for SMAD1-4. Luciferase activities were measured 48 hours after, using the Dual-Luciferase Reporter Assay System (Promega) and a microplate luminometer, Tecan Infinite 200 PRO (Tecan, Männedorf, Switzerland). The signal of firefly luciferase was normalized to the total protein concentration of each well with the control condition set as one. All experiments were carried out in triplicate wells and repeated three times each triplicate well.

### Proteasome Activity

Peptidase activity of the proteasome was measured by mixing cell lysate with 20 μM fluorogenic peptide Suc-LLVT-AMC (succinyl-Leu-Leu-Val-Tyr-7-amino-4-methylcoumarin) (Calbiochem) as previously described^35^. Fluorescence was quantified using a spectrofluorometer (Fluoroskan Ascent FL, Thermo Labsystem). The results represent three independent experiments with each sample ran in triplicate.

### Statistical analyses

Data are representatives of three independent experiments and are expressed as mean ± standard error of the mean (SEM). P-values were calculated using Student *t*-test to compare control and treated groups and p-values less than 0.05 were considered statistically significant (*P < 0.05; **P < 0.001; ***P < 0.001).

### Gene Expression profiling

For differential expression profiling of siScr and siHs27 treated PC3 cells, total RNA was extracted using TRIzol^®^ and the quality of RNAs were measured using Agilent 2100 bioanalyzer (Agilent, Santa Clara, CA). Samples were prepared following Agilent’s One-Color Microarray-Based Gene Expression Analysis Low Input Quick Amp Labeling v6.0. An input of 100 ng of total RNA was used to generate Cyanine-3 labeled cRNA. Samples were then hybridized on Agilent SurePrint G3 Human GE 8 × 60 K Microarray (AMDID 028004) and arrays were scanned with the Agilent DNA Microarray Scanner at a 3 um scan resolution. Data was processed with Agilent Feature Extraction 11.0.1.1. and processed signal was quantile normalized with Agilent GeneSpring 12.0. Normalized log2 values of YAP/TAZ target genes were compared between siHsp27 and siScr treated cells. All microarray profiling analyses were carried out in triplicate.

## Additional Information

**How to cite this article**: Vahid, S. *et al*. Molecular chaperone Hsp27 regulates the Hippo tumor suppressor pathway in cancer. *Sci. Rep*. **6**, 31842; doi: 10.1038/srep31842 (2016).

## Supplementary Material

Supplementary Information
