# Supplementary material for: Molecular chaperone Hsp27 regulates the Hippo tumor suppressor pathway in cancer
Source: Sci Rep. 2016 Aug 24;6:31842. doi: 10.1038/srep31842 (PMC4995483; doi:10.1038/srep31842)
Supplement: Supplementary Information [file srep31842-s1.pdf]

Molecular chaperone Hsp27 regulates the Hippo tumor suppressor pathway in cancer

Sepideh Vahid[1,2], Daksh Thaper[1,2], Kate F. Gibson[1], Jennifer L. Bishop[1] and Amina Zoubeidi*[1,2]

[1]The Vancouver Prostate Centre, University of British Columbia, Vancouver, British Columbia, Canada

[2]Department of Urologic Sciences, University of British Columbia, Vancouver, British Columbia, Canada.

***Correspondence:**

Amina Zoubeidi, PhD

Department of Urologic science, University of British Columbia

The Vancouver Prostate Centre

2660 Oak Street

Vancouver BC V6H3Z6

Phone: (604) 875-4111 # 68880

Fax: (604) 875-5654

Email: azoubeidi@prostatecentre.com

SUPPLEMENTERY FIGURE AND TABLE LEGENDS

Supplementary Table S1: List of YAP/TAZ gene signature and respective normalized log2 values in si Scr and si Hsp27 treated PC3 cells.

Supplementary Table S2: List of YAP/TAZ target genes tested in The Cancer Genome Atlas database for prostate cancer.

Supplementary Table S3: List of primers used for quantitative (RT)-PCR

Supplementary Figure S1: Hsp27 regulates YAP phosphorylation and YAP/TAZ transcriptional activity and targets in prostate, lung and breast cancer cell lines. (a) Protein expression of Hsp27, p-YAP S127, YAP and vinculin treated with 20 nM of 2 different siRNA for Hsp27 compared to si Scrambled in PC3 cells**. (b)** Relative transcriptional activity of TCF, SMAD1-4 and TEAD1 assessed by luciferase assay in si Hsp27 A549 and MDA-MB-453 cells compared to si Scr (=1), Graph represents pooled data from three independent experiments. **(c)** Relative mRNA expression of Hsp27, YAP, TAZ (WWTR1) and YAP/TAZ target genes in si Hsp27 A549 and MDA-MB-453 cells compared to si Scr (=1). Graphs are representative of three independent experiments.

Supplementary Figure S2: MST1 regulates YAP phosphorylation and activity in PC3 cells. (a) Protein expression of Hsp27, MST1, p-YAP S127, total YAP and vinculin in PC3 cells transfected with MST1 (WT MST1) and vector control (mock). **(b)** Relative activity of TCF, SMAD1-4 and TEAD1 assessed by luciferase assay in PC3 cells transfected with MST1 compared to vector control (mock=1). Graph represents pooled data from three independent experiments.

Supplementary Figure S3: Hsp27 knockdown does not affect the expression of other major chaperons in prostate, lung or breast Cancer. (a) Protein expression of Hsp90, Hsp70, Clusterin, Hsp27 and vinculin in PC3, A549 and MDA-MB-453 cells transfected with si

Hsp27 or si Scr. **(b)** Protein expression of Hsp27, Hsp70, Hsp90 and vinculin in MEF HSF1$^{-/-}$ cells with Hsp27 overexpression. PC3 cells were used as positive control for mentioned heat shock proteins. **(c))** mRNA expression of Hsp70 (HSPA1A) and YAP/TAZ target genes (see **Supplementary Table S2**) obtained from the Prostate Cancer TCGA data set.

Supplementary Figure S1

a

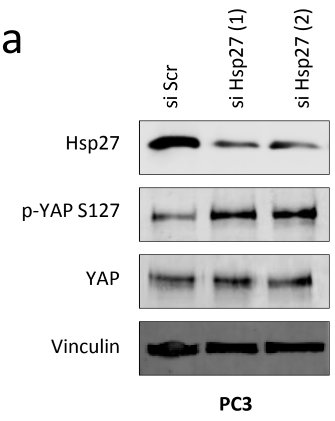

b

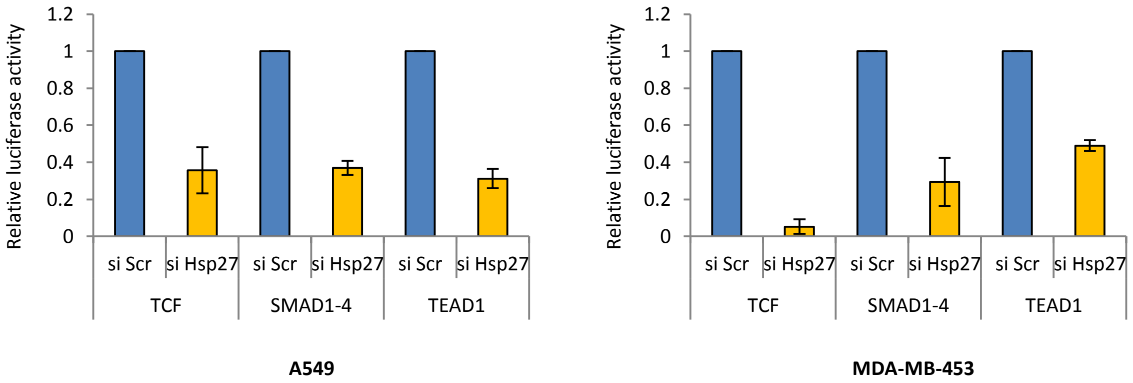

c

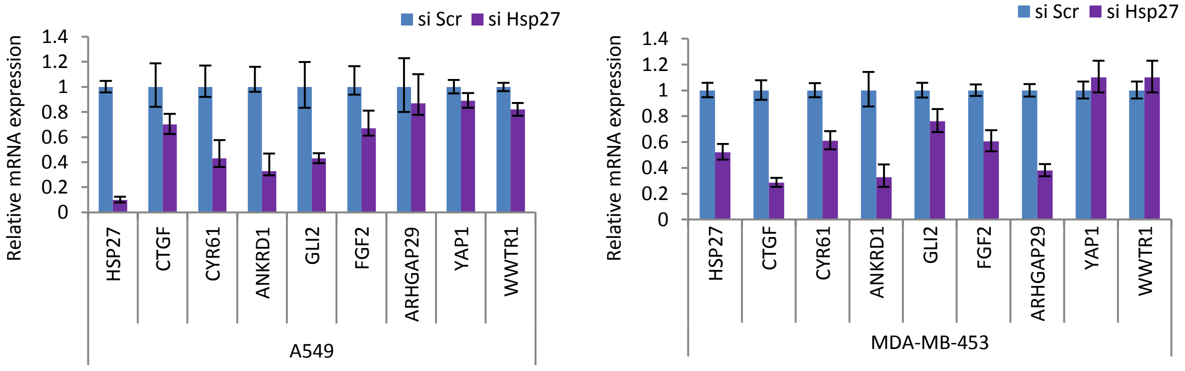

Supplementary Figure S2

a

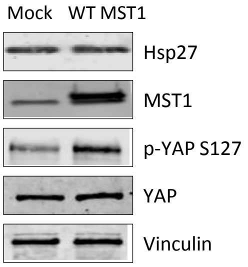

PC3

b

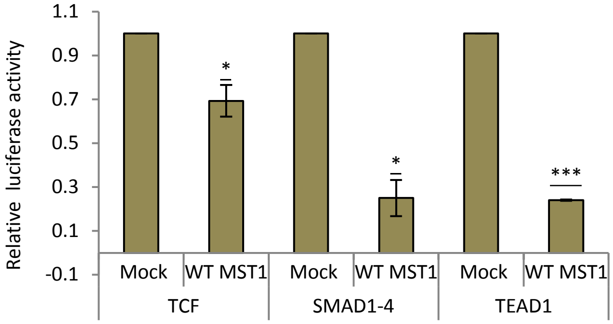

PC3

Supplementary Figure S3

a

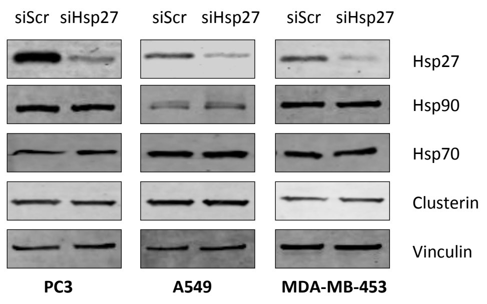

b

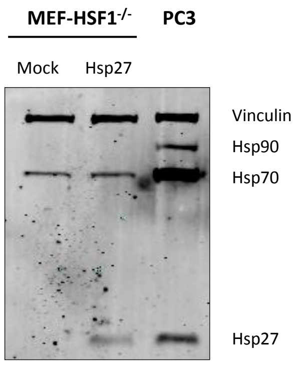

c

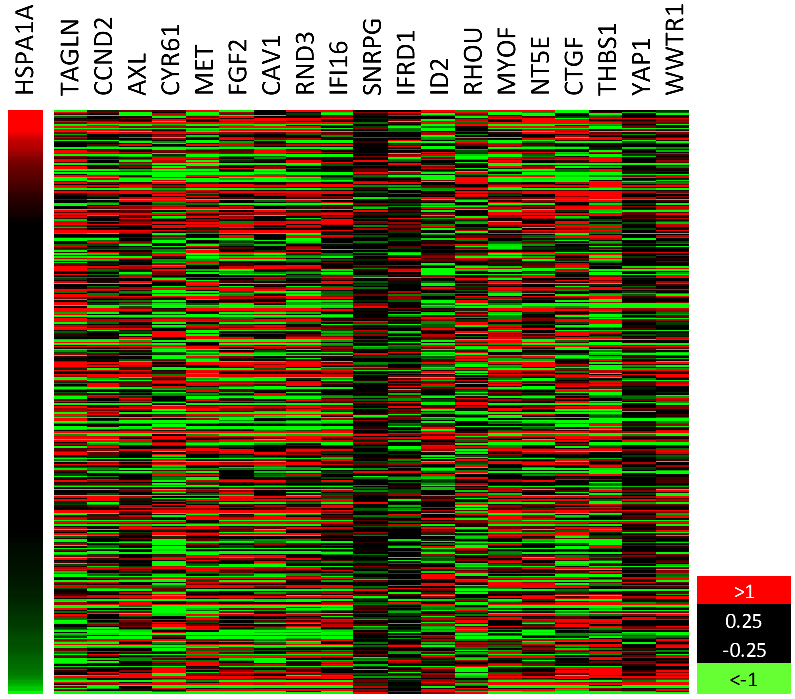

Supplementary Table S1

Gene	si Scr	si Hsp27
PSAT1	17.01364523	16.1642907
SMAD1	15.77624513	14.04485355
PDCD1LG2	15.41523747	13.03842473
MSMO1	14.44154268	13.5796855
SMAD6	14.09740834	13.86459485
SCHIP1	13.83917042	11.42588929
LGALSL	13.48697463	12.36662794
MACC1	13.38562382	11.33284504
UGCG	13.35147678	11.49528249
EPB41L2	13.13598257	10.42346027
PITX2	13.08989616	11.32308609
DAB2	12.73120113	9.442547146
CPA4	12.6546593	11.14469644
SGK1	12.45230107	10.3728517
GPCPD1	12.42749121	9.09193641
PYGO1	12.42169003	8.412915021
CENPF	12.25494483	7.459307484
NT5E	12.09808253	8.255554475
ANXA1	12.00732237	6.890668068
MID1	11.77436835	8.955273931
CHST9	11.55329618	11.39165048
CAV1	11.51225568	7.556603253
PCLO	11.43639944	7.630748029
ARHGAP29	11.43577666	6.087261559
ENC1	11.2918425	9.665932932
TGFA	11.22669414	10.22174955
PRSS23	11.17193986	7.576703769
SERPINB7	11.17093091	9.214762959
MYBL1	10.96697517	8.530321332
BCL2	10.94477655	6.761392428
SERPINE1	10.93055913	8.142799633
THBS1	10.92734813	9.371137888
RND3	10.84163479	5.350830328
CTNNB1	10.80275149	9.240144947
CTNNAL1	10.79686734	6.947355114
EP300	10.71243002	7.898946112
CRIM1	10.63554954	8.386907855
SHROOM3	10.41196544	8.431558379
FRZB	10.40232472	7.362189017
SLIT2	10.35295578	7.58824035
PTPN14	10.27103809	6.666341606
EMP2	10.2659359	9.57971603
SNAPC1	10.26458358	7.145387872

SCD5	10.22401325	7.076027669
MYOF	10.14549825	7.461591073
BICC1	10.10906223	7.341722295
TOP2A	10.08226216	4.789844276
CDH2	9.911701168	7.153638113
LHFP	9.883599282	6.750840879
OPN3	9.814634293	8.469460352
SNAI2	9.583307336	6.189412359
CCND2	9.554704646	7.913117608
ESM1	9.498819395	4.834771394
TGFB2	9.360629972	6.459271661
LRP6	9.21220502	5.925356234
RIMKLB	9.183667138	5.320722922
ID2	9.178417119	4.52703121
TSC22D2	9.177835418	5.627012519
MET	8.949237843	3.821837258
AHNAK	8.898692163	6.254458186
ECT2	8.806316323	3.835926993
GGH	8.804056141	4.43331799
LMBRD2	8.700756036	5.765337189
TMEM154	8.62234022	5.528784582
ARHGEF28	8.599561719	6.656254082
LUM	8.468867234	5.038729883
CCL28	8.467728622	6.398733417
ADAMTS12	8.441855426	6.549500705
ITGBL1	8.376938801	2.12801484
PRRG1	8.352987282	2.224120624
MDFIC	8.345931973	2.094483875
IFIT2	8.197555527	2.264627753
TMEM27	8.177187891	3.354978572
F3	8.115716005	6.928779886
GLS	8.045898437	3.696395629
PDP2	8.011860896	6.620456942
ACSL4	7.933785474	2.662645565
GADD45A	7.701168511	6.037925031
IFI16	7.622054602	2.050958104
FGF2	7.511950981	1.995879439
SDPR	7.462854366	4.965308957
CLDN1	7.239845759	5.320962217
FSTL1	7.083340184	5.046671197
SCML1	7.074799546	1.903302376
EXPH5	7.039980488	4.218589926
AXL	6.945869383	5.133898067
HMMR	6.936588828	2.002870378
PMAIP1	6.880130696	4.199617898
SEMA3C	6.734954858	1.896036602
PHLDA1	6.70416787	3.880824834

IRS1	6.253338848	3.421702896
DAAM1	6.192013225	2.476968698
FST	6.04905035	4.96256993
ASAP1	6.010676018	2.918076275
INSIG1	5.996509531	3.41756635
RHOU	5.991762634	3.120563863
BDNF	5.957348829	2.186108342
HMGCS1	5.844367976	2.159839427
IDI1	5.784216829	3.639358338
CYP1B1	5.763458318	3.870782342
AOX1	5.760320681	5.30405597
SNORA75	5.739108559	3.52797127
PSG5	5.650150278	2.265399203
JPH1	5.603233315	3.438913103
F2RL1	5.597450724	4.187126117
DIXDC1	5.574666968	3.871582299
SP1	5.564356308	3.687062782
SMAD5	5.327253844	4.402483257
LPIN1	5.295987629	2.184630145
PRICKLE1	5.184416781	4.49580554
SNRPG	5.179853968	2.054330556
DDAH1	5.169179416	2.620085155
FSCN1	5.131463136	5.427053199
DUT	4.8357267	2.088257542
EMP1	4.807664862	2.510870108
LCP1	4.683229889	2.353511348
RAB3B	4.467957492	3.081287668
CTGF	4.462551023	4.503400582
SMAD3	4.38810679	4.10214715
S1PR1	4.174602962	2.572433119
FBXW11	3.958380475	2.234871706
IFRD1	3.725963596	1.924834297
CD55	3.711125816	1.916858113
ANXA3	3.627528632	1.996193033
TFPI2	3.607848585	3.986906103
AMPH	3.573904048	2.29338142
TSC22D1	3.569048688	2.286186518
SYT14	3.565704521	1.962384886
ERRFI1	3.511753982	2.239459703
RGS4	3.509068722	2.251786479
SQLE	3.440047241	2.142507146
CSNK2A1	3.397488108	2.125744639
PMP22	3.382857986	2.074582587
GLI2	3.376551556	2.108634709
ADAMTS1	3.371090433	2.62306717
ADAMTS5	3.368382694	2.130463824
SLC2A3	3.366874704	2.221180596

ADRB2	3.351806111	2.072713945
HEXB	3.351496604	2.054180811
GCNT4	3.338375777	2.085630121
SCD	3.283995386	2.003441001
IL8	3.28391955	2.046099953
COX6C	3.261657177	2.024147335
NT5DC3	3.240491564	1.977508755
STXBP1	3.234171572	2.016912687
SLC16A6	3.226513616	2.035211092
KIT	3.222433331	2.037982755
AREG	3.202016281	2.010265667
KRT5	3.177729672	1.961008123
SOX9	3.137527623	1.921058386
MYC	3.105650424	1.882593394
UPK1B	3.088504261	1.863256292
NID2	3.086198317	1.863334638

Supplementary Table S2

Gene name
TAGLN
CCND2
AXL
CYR61
MET
FGF2
CAV1
RND3
IFI16
SNRPG
IFRD1
ID2
RHOU
MYOF
NT5E
CTGF
THBS1

Supplementary Table S3

List of SYBR Green Primers for qRT PCR

Name	Primers Sequences	
	Forward	Reverse
Hsp27	TCCCTGGATGTCAACAACTTC	TCTCCACCACGCCATCCT
YAP1	GACACATGCACCGGAAATTTC	TTGCTGGACGTTTGTTCATCTT
WWRT1	GTATCCCAGCCAAATCTCGTGATG	CAGCGCATTGGGCATACTCATG
MST1 (STK4)	CTGTGGGGCTGGTTCTGTAT	TGTGGGAGGAGGGTTTGTAG
MST1 (Mus Musculus)	TCATTCGGCTACGGAACAAGA	GACCTGCGACTCCAAAGTCTG
CTGF	TTG GCC CAG ACC CAA CTA	GCA GGA GGC GTT GTC ATT
CYR61	AGCCTCGCATCCTATACAACC	TTCTTTCACAAGGCGGCACTC
GLI2	TGGCCGCTTCAGATGACAGATGTTG	CGTTAGCCGAATGTCAGCCGTGAAG
FGF2	TGGTATGTGGCACTGAAACGA	TTCTGCCCAGGTCCTGTTTT
ARHGAP29	TTGGAATTCAGGAGTTCATGC	TCCAAGTAGAGGCTGCACA
ANKRD1	AGCCCAGATCGAATTCCGTG	CTCCTTCTCTGTCTTTGGCGT
GAPDH	ACCCAGAAGACTGTGGATG	CAGTGAGCTTCCCGTTCA
